# A hierarchical Bayesian model to estimate the unobservable predation rate on sawfly cocoons by small mammals

**DOI:** 10.1002/ece3.1394

**Published:** 2015-01-15

**Authors:** Panisara Pinkantayong, Satoshi Suzuki, Mamoru Kubo, Ken-ichiro Muramoto, Naoto Kamata

**Affiliations:** 1 The University of Tokyo Forests, Graduate School of Agricultural and Life Sciences, The University of TokyoTokyo, Japan; 2 The University of Tokyo Chichibu Forest, Graduate School of Agricultural and Life Sciences, The University of TokyoChichibu, Japan; 3 Department of Electrical and Computer Engineering, Graduate School of Natural Science and Technology, Kanazawa UniversityKanazawa, Japan; 4 Ishikawa National College of TechnologyTsubata, Ishikawa, Japan

**Keywords:** *Apodemus argenteus*, *Apodemus speciosus*, cocoon dynamic models, *Myodes rufocanus bedfordiae*, predation rate estimation

## Abstract

Predation by small mammals has been reported as an important mortality factor for the cocoons of sawfly species. However, it is difficult to provide an accurate estimate of newly spun cocoons and subsequent predation rates by small mammals for several reasons. First, all larvae do not spin cocoons at the same time. Second, cocoons are exposed to small mammal predation immediately after being spun. Third, the cocoons of the current generation are indistinguishable from those of the previous generation. We developed a hierarchical Bayesian model to estimate these values from annual one-time soil sampling datasets. To apply this model to an actual data set, field surveys were conducted in eight stands of larch plantations in central Hokkaido (Japan) from 2009 to 2012. Ten 0.04-m^2^ soil samples were annually collected from each site in mid-October. The abundance of unopened cocoons (*I*), cocoons emptied by small-mammal predation (*M*), and empty cocoons caused by something other than small-mammal predation (*H*) were determined. The abundance of newly spun cocoons, the predation rate by small mammals before and after cocoon sampling, and the annual rate of empty cocoons that remained were estimated. A posterior predictive check yielded Bayesian *P*-values of 0.54, 0.48, and 0.07 for *I*, *M*, and *H*, respectively. Estimated predation rates showed a significant positive correlation with the number of trap captures of small mammals. Estimates of the number of newly spun cocoons had a significant positive correlation with defoliation intensity. These results indicate that our model showed an acceptable fit, with reasonable estimates. Our model is expected to be widely applicable to all hymenopteran and lepidopteran insects that spin cocoons in soil.

## Introduction

Accurate estimates of animal abundance provide basic but important information for ecological issues. Ecologists usually use abundance to parameterize predator–prey models or determine species–habitat relationships. In contrast, wildlife managers use abundance to decide management options for pest populations or rare species. Obtaining accurate and reliable methods for estimating abundance is, thus, critical for the success of a diverse array of ecological applications (Conn et al. 2006).

Sawflies are primitive Hymenoptera that are a major taxonomic group of the free-living defoliator guild (Wagner and Raffa 1993). The populations of some sawfly species sometimes reach outbreak level, causing the conspicuous defoliation of host plants. The cocoon stage of these species has received more detailed investigation than the adult, egg, and free-living larval stages for several reasons. Basically, cocoons are easy to sample, incubate, and manipulate (Lejeune 1955; Ives and Turnock 1959). Furthermore, the cocoon stage is thought to be important in determining the population dynamics of sawflies, because a number of mortality factors (such as diseases, invertebrate predators, and vertebrate predators) affect survival during the cocoon stage. Among these factors, predation by small mammals often represents a major source of mortality (Graham 1928; Holling 1959; Hanski and Parviainen 1985).

Several methods have been used to estimate the predation of sawfly cocoons by small mammals (mice, rodents, shrews, and so on), including cocoon planting, cocoon sampling, and the protection of cocoons from mammal predation in forests (Buckner 1959). However, none of these methods could estimate small-mammal predation precisely. Cocoon planting changes the local density and distribution pattern of cocoons, which strongly influences the response of small mammals. Cocoon sampling cannot provide a precise estimate of the number of newly spun cocoons or of cocoons preyed on by small mammals (Buckner 1959). This issue exists because sawflies are exposed to small-mammal predation during the cocoon stage, with the timing of cocoon spinning and adult emergence greatly varying within populations (Turnock 1960). Furthermore, the cocoons of the current generation cannot be distinguished from older ones by the naked eye (Ives 1976). Molecular analysis could be used to determine the freshness of the cocoons (Munch et al. 2008). However, it would be expensive and time-consuming to conduct this technique on thousands of empty cocoons.

Life table analysis is useful for evaluating whether a population is decreasing or increasing and for evaluating the relative importance of each mortality factor with changing population dynamics. This study aimed to develop a hierarchical Bayesian model to estimate the number of newly spun cocoons and the predation rate of the cocoons by small mammals, with this information being important for life table analysis of cocoon-forming insects.

## Materials and Methods

### Model species

*Pristiphora erichsonii* (Hymenoptera: Tenthredinidae) is a monophagous folivorous insect that feeds on larch needles. This species is widely distributed in the northern hemisphere, in which the host tree species (larch, *Larix* spp.) are distributed. There are many reports of population outbreaks of *P. erichsonii*, especially in North America and Japan (Reeks 1954; Turnock 1960; Nairn et al. 1962; Ives 1976). Population outbreaks tend to occur in mature forests and continue for 6–10 years in a given stand (Kamata 2002; Lynch 2012). The feeding period of *P. erichsonii* extends from mid-July to early September in Hokkaido, Japan (P. Pinkantayong, pers. obs.). Fully grown larvae drop to the soil surface and spin cocoons in the soil, where they overwinter (Lejeune 1947). Prepupae and pupae of *P. erichsonii* spend approximately 10 months inside a cocoon, even though the pupal period lasts for about 1 week only before adult emergence. However, the timing of cocoon spinning and adult emergence varies greatly among years and individuals, even at the stand level (Turnock 1960).

### Study sites

**Figure 1:**
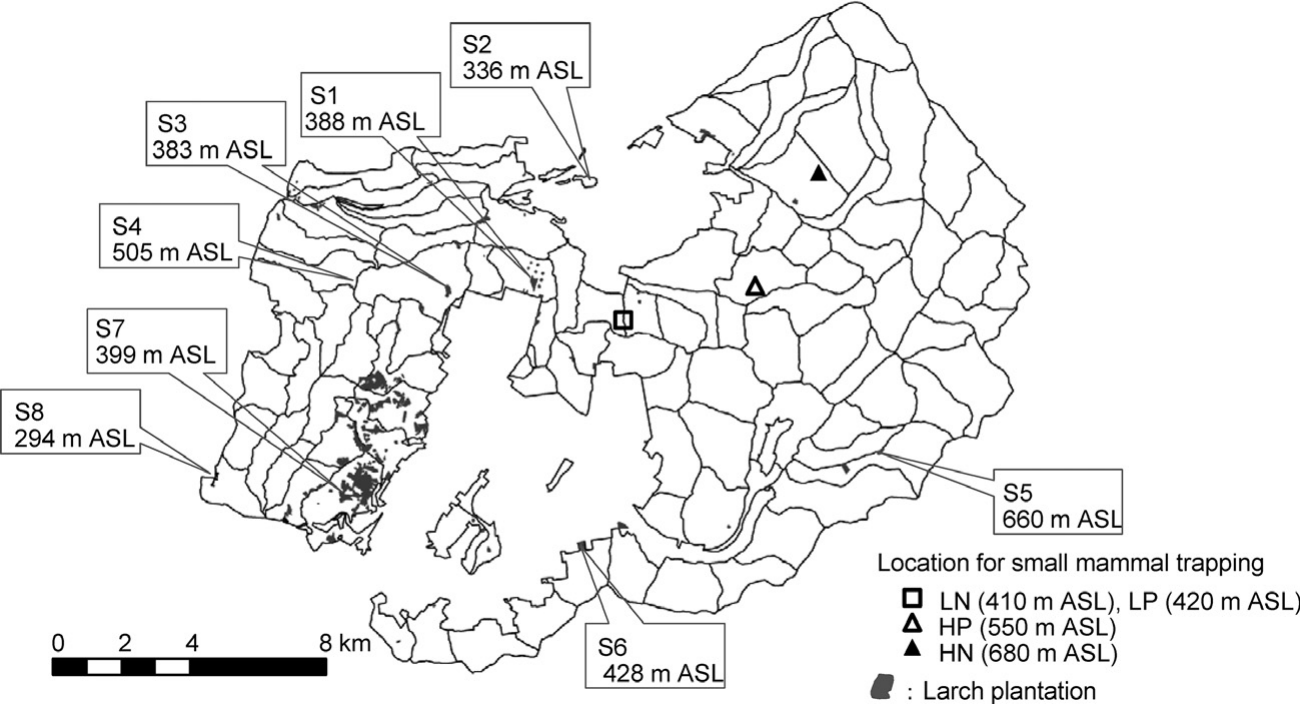
Locations of the eight study sites used for cocoon sampling and the four stands used for small mammal trapping in the University of Tokyo Hokkaido Forest in central Hokkaido, Japan.

This study was carried out in the University of Tokyo Hokkaido Forest (UTHF) (142°18′–40′E, 43°10′–20′N), which is located in central Hokkaido, Japan (Fig.1). Small patches of larch plantations (158.48 ha in total; representing 0.7% of total UTHF area) are scattered in the UTHF territory (22,733 ha of total forest area) (The University of Tokyo Forest 2012). Cocoon sampling was conducted at eight larch plantation stands of *Larix kaempferi* (also known as *Larix leptolepis*), with the exception of site 4, in which *Larix gmelinii* (also known as *Larix dahurica*) and a hybrid of both larch species (*L. gmelinii *× *L. kaempferi*) were planted. The altitudes of these sites range from 294 to 660 m above sea level (a.s.l.). The distance between any two of the eight sites ranged from 2.6 to 19.4 km. Small-mammal trapping was conducted at two stands of a *Picea glehnii* plantation and two stands of natural boreal forest containing natural and broadleaved and coniferous trees (410–680 m a.s.l.). All four stands differed to the other locations used for cocoon sampling. The two *Picea* stands were located at two slightly different altitudes (LP, 420 m a.s.l.; HP, 550 m a.s.l.). The two natural forests were also located at two slightly different altitudes (LN, 410 m a.s.l.; HN, 680 m a.s.l.). The distance between any two of the four trapping sites ranged from 50 m (LP–LN) to 7.2 km (LN–HN). The distance from the trapping sites to the cocoon sampling sites ranged from 2.9 to 19.8 km.

In Furano City, where the UTHF is located, population outbreaks of *P. erichsonii* have occurred every year since 2008. In the UTHF, severe defoliation was first recorded in 2009.

### Cocoon sampling

**Figure 2:**
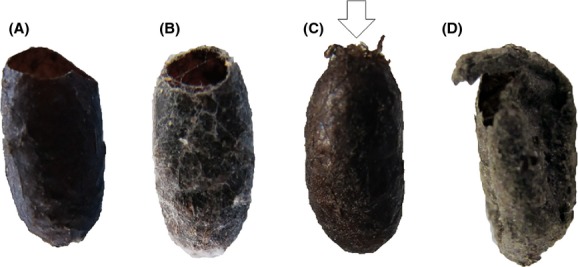
The different appearances of the hole in *Pristiphora erichsonii* (Hartig) cocoons caused by: (A) normal emergence of a *P. erichsonii* adult, (B) parasitic wasp, (C) parasitic fly, and (D) predation by small mammals.

Cocoons were sampled in mid-October of 2009–2012, because sampling needed to be completed before snow cover but after all larvae had spun cocoons. The October sampling enabled us to determine the cocoon predation rate by small mammals before and after overwintering, separately, which was useful for life table analysis. In this paper, we did not mention mortality by parasitoids because it was irrelevant to the hierarchical Bayesian model. However, the October sampling also enabled us to determine mortality by each parasitoid species through incubating sampled intact cocoons because all parasitoids emerged after overwintering (P. Pinkantayong, pers. obs.). Ten topsoil samples (20 × 20 cm each, with approximately 15 cm depth) were collected, because it has been reported that *P. erichsonii* mature larvae enter the soil and spin cocoons at depths shallower than 7 cm (Higashiura and Suzuki 1981). The topsoil samples were collected from each site, according to a specific methodology. In 2009, five sample trees were selected arbitrarily in each site. The same sample trees were then used throughout this study. The distance between the two nearest sample trees was approximately 4–8 m. Two topsoil samples were collected from under the canopy of each of the sample trees. The distance between any two samples below one tree was approximately 1–5 m. When sampling the soil, the sites of former sample collections were avoided because the abundance of old cocoons would be underestimated. The collected soils were then transferred to a nursery of the UTHF. Cocoons of *P. erichsonii* were then manually collected by sieving and hand sorting. The cocoons were separated into three categories: unopened healthy-looking cocoons with a healthy larva or a parasitized larva (hereafter referred to as “unopened cocoons”), empty cocoons caused by small-mammal predation, and empty cocoons caused by something other than small-mammal predation. For cocoons with a hole, the cause was judged on the basis of the appearance of the hole (Krause and Raffa 1996): (1) a smooth and spherical exit hole at the end of the cocoon indicated the normal emergence of a *P. erichsonii* adult, (2) a small and slightly jagged spherical exit hole near the end of the cocoon indicated a parasitic wasp, (3) a very small nonspherical exit hole at the end of the cocoon indicated a parasitic fly, and (4) a jagged or shredded opening of variable size indicated small-mammal predation (Fig.2) (Buckner 1956; Dahlsten 1967; Ives 1976). Because predation by small mammals occurs during the period immediately after the cocoon is spun until adult emergence, the cocoons preyed on by small mammals from the soil samples included cocoons of both the current generation and the previous ones, which were indistinguishable from each other by the naked eye (Ives and Turnock 1959; Ives 1976). Another category, “empty cocoons caused by something other than small-mammal predation” included the following: the normal emergence of a *P. erichsonii* adult, parasitoid emergence, and those killed by diseases. This category only included previous generations and excluded the cocoons of the current generation.

### Hierarchical Bayesian model of cocoon dynamics

**Figure 3:**
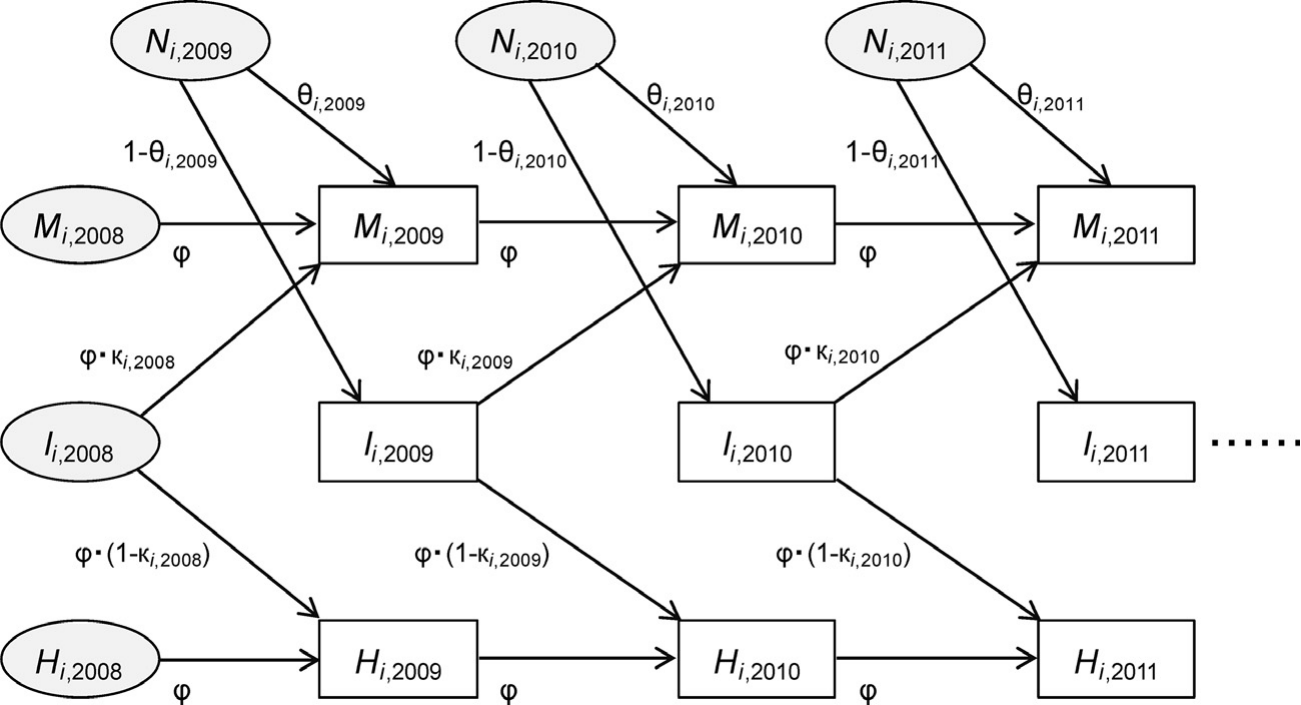
Cocoon dynamics model. *N*_*i*, *t*_, newly spun cocoons in the summer of generation *t*; θ_*i*, *t*_, predation rate by small mammals before October sampling of generation *t*;*κ*_*i*, *t*_, predation rate by small mammals after October sampling of generation *t*; φ, annual remaining rate of empty cocoons; *M*_*i*, *t*_, empty cocoons due to small-mammal predation in October samples of year *t* including previous generations; *I*_*i*, *t*_, unopened cocoons spun in year *t* and found in October samples of the same year, which include healthy-looking cocoons and the current generation's cocoons with mycelia; *H*_*i*, *t*_, cocoons emptied by something other than small-mammal predation in the October samples of year *t*, which consisted of previous generations.

In this study, we developed a model to describe cocoon dynamics based on a Bayesian approach to estimate: (1) the abundance of the cocoons in the current generation before mammal predation, (2) predation rate during the period from cocoon spinning to sampling in October (hereafter referred to as “predation rate before October sampling”), and (3) the predation rate from sampling in October to adult emergence the following summer (hereafter referred to as “predation rate after October sampling”) (Fig.3). Small mammals prey on unopened cocoons without discriminating between healthy cocoons and cocoons parasitized by parasitoids (Hardy 1939). Thus, an identical parameter was used for each of the two predation rates before and after October sampling. The hierarchical model consists of data models and process models. The data models relate to the observed number of cocoons in sample *j* at site *i* in year *t* and to the expected number of cocoons in the same sample (λ_*I,i,j,t*_, λ_*M,i,j,t*_, and λ_*H,i,j,t*_) in which the observed cocoons are unopened cocoons (*I*_*i,j,t*_), empty cocoons due to small-mammal predation (*M*_*i,j,t*_), and empty cocoons caused by something other than small-mammal predation (*H*_*i,j,t*_). Poisson models were used because the number of cocoons is count data, whereby: 

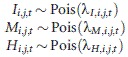


The process models describe how the numbers of cocoons from each category in each year are related to each other. λ_*I,i,j,t*_ is the number of cocoons spun in the summer of year *t* and not preyed on by small mammals by the time of sampling in October. λ_*M,i,j,t*_ is the sum of the following: cocoons spun in the summer of year *t* but subjected to small-mammal predation by the October sampling of year *t*, cocoons spun in the summer of year *t*−1 but subjected to small-mammal predation in the summer of year *t*, and cocoons spun in the summer before year *t*−1 but subjected to small-mammal predation in the summer of the year before *t*−1. λ_*H,i,j,t*_ is the sum of empty cocoons caused by something other than small-mammal predation, which were spun before year *t*. The model formulae are as follows: 

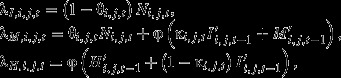
where θ_*i,j,t*_ and κ_*i,j,t*_ are the predation rate before October sampling and the predation rate after October sampling in sample *j* at site *i* in year *t*, respectively. *N*_*i,j,t*_ is the number of cocoons spun in year *t* in sample *j* at site *i* in year *t*. φ is the annual remaining rate of empty cocoons, which ranged from 0 to 1. We assumed that the prior distribution of φ is a standard uniform distribution, U(0,1). *I’*_*i,j,t*−1_, *M’*_*i,j,t*−1_, and *H’*_*i,j,t*−1_ are latent variables for the number of unopened cocoons, opened cocoons (due to small-mammal predation), and empty cocoons (caused by factors other than small-mammal predation), respectively, from the previous year (*t*−1) that were already present in the soil samples of the current year (*t*).We used informative prior distributions for the parameters and gamma distributions with the same mean and variance as the observed distributions of *I*_*i,j,t*−1_, *M*_*i,j,t*−1_, and *H*_*i,j,t*−1_. Of note, this is an important component of the Bayesian model because we were unable to determine how many cocoons from previous years remained in the soil samples of the current year directly, which represented an important limitation. This approach does not lead to the duplicated use of data because *I’*_*i,j,t*−1_, *M’*_*i,j,t*−1_, and *H’*_*i,j,t*−1_ are used to estimate the number of cocoons in the current year (λ_*I,i,j,t*_, λ_*M,i,j,t*_, and λ_*H,i,j,t*_), but not in the previous year (λ_*I,i,j,t*−1_, λ_*M,i,j,t*−1_, and λ_*H,i,j,t*−1_). θ_*i,j,t*_ and κ_*i,j,t*_ were assumed to follow beta distributions Be(α = k_θ_*p*_θ,*i,t*_, β = k_θ_(1 - *p*_θ,*i,t*_)) and Be(α = k_κ_p_*κ*, *i,t*_, β = *k*_κ_(1 - *p*_κ,*i,t*_)), respectively, where *p*_θ,*i,t*_ and *p*_*κ*, *i,t*_ are the mean predation rate before October sampling and the mean predation rate after October sampling at site *j* in year *t*, respectively. *k*_θ_ and *k*_κ_ are parameters that determine the dispersion of the distribution, and we assumed an informative Gamma(α = 1, β = 1) prior, where α and β are the shape and rate parameters. Because *N*_*i,j,t*_ is a positive continuous variable, it is assumed to be distributed following a gamma distribution, Gamma(α = N_*i,t*_*/s*, β = 1/*s*), where *N*_*i,t*_ is the mean number of cocoons of the generation in year *t* per sample at site *i* in year *t*. Noninformative priors are used for hyperparameters. For instance, a uniform distribution U(0, 1) is used for parameters between 0 and 1, φ, *p*_θ,*i,t*_, and *p*_κ,*i,t*_. In comparison, an inverse gamma distribution, InvGamma(α = 0.001,β = 0.001), is used for parameters >0,*s*, *N*_*i,t*_, k_*θ*_, and k_*κ*_, where α and β are the shape and scale parameters, respectively. The total predation rate throughout the cocoon period (hereafter referred to as the “total predation rate”) (ρ) was obtained by the following equation: 

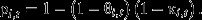


The random walk Metropolis–Hasting algorithm (Metropolis et al. 1953; Hastings 1970), which is a Markov chain Monte Carlo (MCMC) method, was used to sample from the posterior distributions of model parameters. We wrote the MCMC sampling algorithm in C. We ran three MCMC chains for 5 × 10^5^ iterations, with a burn in of 2 × 10^5^ iterations. The MCMC samples were thinned to one every 100th sample, to reduce autocorrelation and due to computational limitations. Convergence of the MCMC was checked by the Gelman–Rubin statistic. The 95% credible intervals (CIs) of the posterior distribution of parameters were calculated from the 95% highest posterior probabilities using the “HPD interval” function from the “coda” library (Plummer et al. 2012) in R ver. 2.15.3 (R Development Core Team 2013).

To assess the fit of the model, we computed the posterior predictive *P*-value using deviance as a test quantity (Gelman et al. 2004), where an extreme *P*-value (smaller than 0.05 or >0.95) indicates a large discrepancy between the actual data and the model.

### Small mammal data

We used small-mammal trapping data obtained from the regular monitoring of forest pests by the UTHF, which was authorized by the Kamikawa Branch Office, Hokkaido Prefectural Government under the Wildlife Protection and Proper Hunting Act (http://law.e-gov.go.jp/htmldata/H14/H14HO088.html; Ministry of the Environment, Japan). Small-mammal trapping was conducted in early June (spring) and mid-September (autumn) of every year since 2000. We used the data from June 2009 to June 2013. Fifty snap traps (PANCHU®, Otsuka, Osaka, Japan) containing peanuts as bait were deployed in two 5 by 5 grids at LP and LN sites and in 5 by 10 grids at the HP and HN sites. The grid spacing in both cases was 10 m. The traps were set for three nights for each survey. The traps were checked daily in the morning. Captured mammals were transferred to the laboratory and were identified to the taxa level. Predominant taxa included the small Japanese field mouse (*Apodemus argenteus*), the large Japanese field mouse (*Apodemus speciosus*), the grey red-backed vole (*Myodes rufocanus bedfordiae*), and shrews (*Sorex* spp.). The PANCHU® is a type of snap trap that kills a small mammal within 3 min and conforms to the “Guidelines of the American Society of Mammalogists for the use of wild mammals in research (Sikes and Gannon 2011)”. No red data species have been collected since 2000. The total number of captures per 100 traps per three nights was used for in the analyses.

### Posterior statistical analysis

For a validity test from a biological viewpoint, relationships between the trap captures of small mammals and the three predation rates (θ, κ, and ρ) were examined using a beta regression, with the trap captures being used as an explanatory variable and predation rates as a response variable. The autumn trap captures were used for the predation rate before the October sampling (θ). The spring trap captures were used for the predation rate after the October sampling (к). The average of autumn captures in a given year and spring captures in the following year were used to calculate the total predation rate (ρ). The three relationships were obtained using the total trap captures of the three major species (*A. argenteus*, *A. speciosus*, *M. r. bedfordiae*) by 100 traps at four trapping sites as an explanatory variable. *Sorex* spp. were excluded from the analysis because of the small number of captures of these species. The raw trap capture data are presented in Table S1. The coefficient and its *P*-value were determined. A “betareg” library (Zeileis et al. 2013) in R ver. 2.15.3 was used for the beta regression.

Defoliation intensity was compared with the number of spun cocoons (*N*) and observed intact cocoons (*I*). For seven sites (excluding site 4), defoliation data were cited from Pinkantayong et al. (2015). Sites 5–8 in this study corresponded to sites 4–7 in Pinkantayong et al. (2015). At site 4, defoliation intensity was obtained from canopy photographs, following the same methodology as Pinkantayong et al. (2015).The average percentages of defoliation intensity are presented in Table S2. Pearson's correlation coefficient was determined between defoliation intensity and each of the two cocoon abundance types (*N* and *I*) by including/excluding the results of site 5, where cocoon abundance was low, even in years with severe defoliation.

## Results

The abundance of unopened cocoons (*I*) in the field data peaked in 2010 or 2011, depending on the site, and decreased in six sites in 2012. The observed abundance of *M* and *H*, which were influenced by both the current and the previous generations of cocoons, continued to increase in 2011 and 2012. In comparison, the observed *I*, which was only influenced by the current generation, decreased greatly at many sites in 2012 (Table1). The model estimated the annual remaining rate of cocoons (φ) at 0.743 (95% CI, 0.722–0.768; Table2). The estimated abundance of *N* fluctuated in a similar manner to the observed *I* in the model. The modeled predation rate before October sampling (θ) was prone to increase with each year. There was no constant tendency in the rates of predation by *M* between the period before and after October sampling (θ and κ). Total mortality (ρ) was low in 2009, but was higher at sites 6 and 7 compared to the other sites. Mortality increased during 2010 and 2011. The posterior predictive *P*-values indicate that our model adequately explained the collected field data (0.54 for *I*, 0.48 for *M*, and 0.07 for *H*).

**Table 1:** Abundance of unopened cocoons of *Pristiphora erichsonii* (Hartig) (*I*), empty cocoons preyed on by small mammals (*M*), and cocoons emptied by something other than predation by small mammals (*H*) observed in soil samples collected from October 2009 to October 2012 at eight larch plantations in the University of Tokyo Hokkaido Forest

		Site								
Category	Generation	1	2	3	4	5	6	7	8	Average
*I*	2009	6.6 (3–15)	5.0 (1–10)	13.6 (0–60)	8.0 (0–28)	4.6 (0–20)	47.4 (17–73)	24.1 (11–51)	8.2 (2–27)	14.7
	2010	37.9 (12–107)	35.8 (4–81)	21.5 (5–50)	8.9 (1–18)	1.5 (0–7)	8.3 (3–18)	6.4 (3–18)	29.3 (9–77)	18.7
	2011	14.9 (3–34)	10.0 (3–18)	6.0 (0–19)	1.3 (0–6)	0.3 (0–2)	21.1 (0–66)	20.3 (4–66)	15.0 (6–35)	11.1
	2012	0.4 (0–3)	7.4 (1–23)	3.7 (0–24)	0.0 (0)	0.1 (0–1)	12.6 (4–24)	16.8 (3–56)	2.6 (0–11)	5.45
*M*	2009	0.0 (0)	0.7 (0–3)	0.1 (0–1)	0.0 (0)	0.0 (0)	9.0 (0–25)	3.8 (0–14)	0.0 (0)	1.70
	2010	8.7 (2–18)	6.9 (0–27)	3.9 (0–12)	0.9 (0–5)	1.7 (0–8)	37.6 (17–72)	16.5 (3–37)	3.6 (1–11)	9.98
	2011	37.6 (11–94)	24.4 (2–79)	10.4 (0–21)	1.6 (0–6)	3.6 (0–9)	62.5 (8–102)	18.0 (0–34)	11.8 (2–45)	21.2
	2012	34.5 (2–58)	22.8 (4–77)	14.5 (0–32)	3.8 (0–10)	5.2 (0–24)	60.7 (31–125)	18.1 (5–31)	16.3 (6–29)	22.0
*H*	2009	0.2 (0–1)	0.5 (0–2)	0.4 (0–2)	0.4 (0–1)	0.2 (0–1)	4.5 (2–8)	6.3 (2–16)	0.0 (0)	1.56
	2010	8.6 (4–17)	16.1 (6–27)	20.5 (4–40)	7.5 (0–27)	2.6 (0–10)	25.5 (10–52)	17.9 (3–34)	16.8 (6–30)	14.4
	2011	33.6 (11–73)	24.0 (6–50)	22.5 (7–46)	4.5 (0–14)	2.0 (0–5)	23.8 (6–67)	22.1 (0–57)	16.3 (4–34)	18.6
	2012	29.5 (3–52)	31.3 (9–62)	15.1 (0–55)	2.3 (0–7)	5.5 (0–21)	46.8 (16–60)	24.3 (8–75)	42.6 (27–67)	24.7

Mean (minimum-maximum) (/0.04 m^2^).

**Table 2:** The number of newly spun cocoons (*N*) per 0.04 m^2^, predation rates by small mammals before and after October sampling (θ and κ), and the annual remaining rate of empty cocoons (φ) estimated by a Bayesian hierarchical model from 2009 to 2012 at eight larch plantations in the University of Tokyo Hokkaido Forest. The predation rate throughout the cocoon period (ρ) was estimated from the posterior distribution of θ and κ

		Site							
Parameter	Generation	1	2	3	4	5	6	7	8
*N*	2009	12.67(7.25–19.60)	10.73(5.98–16.90)	9.95(5.18–16.39)	10.78(5.88–17.17)	8.60(4.45–14.27)	59.10(43.58–76.43)	31.92(21.00–44.42)	13.22(7.80–20.17)
	2010	46.97(33.79–61.82)	39.39(27.27–53.74)	27.17(17.80–38.88)	14.01(8.27–21.40)	4.41(1.85–8.23)	25.88(13.99–40.69)	17.15(9.10–28.10)	33.32(22.18–45.78)
	2011	32.59(17.69–51.21)	19.68(11.06–32.06)	10.81(5.67–17.83)	4.34(1.83–8.04)	3.57(1.32–7.53)	38.88(23.82–56.22)	23.36(14.62–35.01)	21.90(13.25–31.92)
	2012	6.23(1.91–15.57)	14.11(7.87–23.34)	6.55(2.98–11.82)	3.62(1.39–7.59)	3.82(1.47–7.64)	27.90(14.43–45.74)	21.15(12.88–31.40)	13.29(5.84–23.23)
θ	2009	0.032(0.010–0.081)	0.048(0.009–0.151)	0.029(0.008–0.076)	0.032(0.009–0.083)	0.044(0.011–0.114)	0.149(0.085–0.225)	0.095(0.037–0.169)	0.028(0.008–0.074)
	2010	0.185(0.108–0.269)	0.132(0.064–0.212)	0.101(0.023–0.197)	0.051(0.011–0.131)	0.194(0.024–0.476)	0.540(0.239–0.725)	0.402(0.080–0.650)	0.104(0.031–0.189)
	2011	0.437(0.115–0.659)	0.256(0.036–0.536)	0.191(0.018–0.464)	0.184(0.024–0.474)	0.716(0.262–0.938)	0.514(0.314–0.663)	0.127(0.022–0.303)	0.082(0.016–0.224)
	2012	0.804(0.276–0.971)	0.266(0.039–0.534)	0.275(0.030–0.620)	0.866(0.417–0.986)	0.844(0.357–0.977)	0.398(0.078–0.650)	0.145(0.020–0.339)	0.705(0.433–0.857)
κ	2009	0.106(0.025–0.281)	0.054(0.017–0.121)	0.092(0.025–0.215)	0.075(0.016–0.193)	0.368(0.120–0.644)	0.454(0.256–0.629)	0.373(0.116–0.604)	0.081(0.020–0.205)
	2010	0.343(0.078–0.590)	0.528(0.274–0.743)	0.395(0.144–0.655)	0.240(0.047–0.525)	0.584(0.109–0.955)	0.520(0.059–0.928)	0.534(0.102–0.941)	0.567(0.272–0.847)
	2011	0.484(0.083–0.902)	0.224(0.035–0.596)	0.838(0.344–0.980)	0.679(0.139–0.966)	0.287(0.021–0.817)	0.174(0.030–0.461)	0.245(0.040–0.583)	0.081(0.016–0.233)
	2012	NA	NA	NA	NA	NA	NA	NA	NA
ρ	2009	0.135(0.046–0.306)	0.100(0.040–0.209)	0.118(0.047–0.243)	0.105(0.035–0.224)	0.396(0.151–0.655)	0.535(0.365–0.690)	0.433(0.190–0.644)	0.107(0.038–0.226)
	2010	0.465(0.249–0.667)	0.590(0.370–0.778)	0.456(0.228–0.689)	0.279(0.079–0.553)	0.665(0.245–0.966)	0.782(0.507–0.968)	0.724(0.350–0.965)	0.612(0.350–0.862)
	2011	0.709(0.373–0.949)	0.424(0.138–0.740)	0.868(0.450–0.985)	0.737(0.275–0.973)	0.798(0.393–0.972)	0.599(0.422–0.763)	0.341(0.111–0.652)	0.157(0.044–0.342)
	2012	NA	NA	NA	NA	NA	NA	NA	NA

NA, not available.

An annual remaining rate of empty cocoons (φ) = 0.743. Posterior mean (95% CIs).

The three predation rates estimated from the model showed significant positive relationships with the number of trap captures of small mammals (Table3). Both *N* and *I* had a significant positive relationship with defoliation intensity (Table4). The relationship was stronger in *N* than in *I* and was stronger when site 5 data were excluded.

**Table 3:** Results of a beta regression determining the effects of total trap captures of three major small mammal species (*Apodemus argenteus*, *Apodemus speciosus*, and *Myodes rufocanus bedfordiae*) on three predation rates (θ = before the October sampling, κ = after the October sampling, and ρ = the predation rate throughout the cocoon period)

Explanatory variable (trap captures)	Response variable (predation rate)	Coefficient	*P*
Autumn	θ	0.021	0.000
Spring	к	0.019	0.017
Average of autumn and the following spring	ρ	0.021	0.007

**Table 4:** Pearson's correlation coefficients of defoliation intensity with the abundance of newly spun cocoons (*N*) and the abundance of observed intact cocoons (*I*) from 2009 to 2012 at eight larch plantations in the University of Tokyo Hokkaido Forest. Results including and excluding data from site 5 are shown

Value	Data from site 5	*r*	*P*	*n*
*N*	w	0.49	0.0046	32
	wo	0.55	0.0020	28
*I*	w	0.44	0.0117	32
	wo	0.50	0.0070	28

## Discussion

To construct a life table, it is important to obtain reliable data about the number of individuals and mortality factors of a population. However, it was difficult to estimate the number of spun cocoons and the predation rate of cocoons by small mammals for several reasons. First, the timing of spinning cocoons varies greatly among individuals in a single stand, with small mammals starting to prey on cocoons soon after cocoons are spun. Second, many empty cocoon shells remain in the soil for long periods of time. Using our model, we estimated annual remaining rate of empty cocoons in the soil to be 74.3%. It is also difficult to distinguish old empty cocoons from the current year empty cocoons by the naked eye. Therefore, in this study, we developed a hierarchical model to estimate the number of newly spun cocoons and the number of cocoons preyed on by small mammals from annual cocoon sampling in October over a 4-year period. Our model provides the number of newly spun cocoons and the cocoon predation rate by small mammals before and after sampling, which improved the quantity and quality of information for life table analysis. Our model was based on the assumption that the remaining rate of empty cocoons was constant among years and locations. We believe that this assumption is reasonable because the decomposition rate of empty cocoons is influenced by certain environmental factors, such as temperature and moisture, including the activity of bacteria. In addition, we assumed that the number of cocoons formed before 2009 was negligible because the value *H*, which was influenced by cocoons that had been spun in the previous year or before, was much smaller in 2009 than in 2012, with the exception of sites 6 and 7 (Table1). When the model was applied to a field data set, this assumption may have acted as a source of error, especially for sites 6 and 7. However, all the posterior predictive *P*-values of *I*, *M*, and *H* in our model were between 0.05 and 0.95, which indicate that the model adequately described the data. The three tested predation rates (θ, к, ρ) were well explained by the number of trap catches of small mammals (Table3). This result also indicates that our model provided reliable estimates of predation rates by small mammals.

Defoliation intensity is strongly influenced by larval density. The abundance of *N* changed in the same manner as the defoliation intensity, with the exception of site 5. Correlations of the defoliation intensity with *N* and *I* were greater when data from site 5 were excluded, rather than included (Table4). At site 5, *N* was small in all 4 years despite intensive defoliation in 2010 and 2012. A large numbers of *P. erichsonii* larvae were crawling on trunks of larch trees in August in both years, even though an epidemic of other defoliators was not documented during this study. This result indicates low survivorship between the late free-living larval stages and the cocoon stage. At site 5 in August 1983, before larch saplings were planted, the top soil was scarified to a depth of 20 cm from the surface by bulldozers (Owari et al. 2011). Thus, the topsoil was still shallow at site 5 during our study (data not shown). The failure of most larvae to spin cocoons at this site was probably due to the shallow topsoil layer. Thus, the defoliation intensity was more strongly correlated with *N* than *I* (Table4). This result indicates that our model provided appropriate estimates, because *I* was determined by *N* and predation before sampling (θ).

In 2012, the values of *M* and *H* were high at four of the sites (sites 1, 2, 3, and 8), even though no conspicuous defoliation was found in the proximate year (Table1). *H* was only influenced by cocoons from previous years. *M* was influenced both by cocoons from previous years and by predation in the current year (Fig.3). Therefore, the high *H* and *M* values at these four sites in 2012 were due to high cocoon density in 2010 and 2011. Prolonged diapause has been reported for *P. erichsonii* (Ives and Turnock 1959); thus, *I* should include prolonged diapause from the previous years. However, prolonged diapause represented <1% of all cocoons (Panisara Pinkantayong, pers. obs.). Therefore, the effect of prolonged diapause was not included in our model. In fact, a significant positive correlation was found between the observed value of *I* and defoliation intensity, indicating that the effects of prolonged diapause were negligible.

The modeling approach presented in this study calculates the level of small-mammal predation of *P. erichsonii* cocoons with high precision. Studies have reported that the cocoons of the current generation are indistinguishable to those of previous generations for many sawfly species, with small-mammal predation starting before the onset of sampling (Morris 1949). Our model is expected to be applicable to many hymenopteran and lepidopteran insects that also spin cocoons and overwinter as cocoons in the soil. However, other aspects of the present study require further investigation. One issue involves improving our model by testing it against a new data set. Furthermore, it would be interesting to compare our model results against other similar modeling approaches. The effects of prolonged diapause also need to be included in the model for certain sawfly species, such as the European spruce sawfly, *Gilpinia hercyniae* (Morris 1949), the brown-headed ash sawfly, *Tomostethus multicinctus* (Cranshaw 2004), and the yellow-headed spruce sawfly, *Pikonema alaskensis* (Katovich et al. 1995). We also believe that our model could be applied to species that overwinter in a stage other than a cocoon, such as *Diprion pini*. Our model could also be expanded to determine the type of small-mammal predation on cocoons, by including small-mammal trapping data into our original model and the functional response of small mammals. The function of the functional response could be improved by minimizing the difference between the estimated values of the original model and the expanded model.

## Supporting Information

**Table S1.** Total number of the three major species (*Apodemus argenteus*, *Apodemus speciosus*, and *Myodes rufocanus bedfordiae*) captured by snap traps in the University of Tokyo Hokkaido Forest from spring 2009 to autumn 2013. **Table S2.** Average percentage of defoliation intensity at eight larch plantations in the University of Tokyo Hokkaido Forest from 2009 to 2012 (some data were extracted from Pinkantayong et al.)
