# Supplementary material for: A hierarchical Bayesian model to estimate the unobservable predation rate on sawfly cocoons by small mammals
Source: Ecol Evol. 2015 Jan 15;5(3):733–42. doi: 10.1002/ece3.1394 (PMC4328775; doi:10.1002/ece3.1394)
Supplement: Supplementary file 1 [file ece30005-0733-sd1.doc]

**Table S1** Total number of the three major species (*Apodemus argenteus*, *Apodemus speciosus*, and *Myodes rufocanus bedfordiae*) captured by snap traps in the University of Tokyo Hokkaido Forest from spring 2009 to autumn 2013.

| Year | Season | *Apodemusargenteus* | *Apodemusspeciosus* | *Myodesrufocanusbedfordiae* |
| --- | --- | --- | --- | --- |
| 2009 | Spring | 15 | 0 | 4 |
|  | Autumn | 23 | 5 | 20 |
| 2010 | Spring | 4 | 1 | 2 |
|  | Autumn | 17 | 2 | 10 |
| 2011 | Spring | 55 | 35 | 70 |
|  | Autumn | 92 | 118 | 63 |
| 2012 | Spring | 162 | 0 | 45 |
|  | Autumn | 138 | 7 | 107 |
| 2013 | Spring | 4 | 0 | 0 |

**Table S2 Average percentage of defoliation intensity at eight larch plantations in the University of Tokyo Hokkaido Forest from 2009 to 2012 (some data were extracted from Pinkantayong *et al*. . At each site, three canopy photos were taken at three points set linearly at 5-m intervals in June and October in both full colour and the near-infrared (NIR) band. The defoliation intensity of each individual tree was obtained by comparing photographs between June and October. Sites 5, 6, 7, and 8 in this table are shown as sites 4, 5, 6, and 7 in the Pinkantayong *et al*. , respectively. Data from site 4 were newly created in this table.**

| Site | Defoliation intensity (%) | | | |
| --- | --- | --- | --- | --- |
|  | 2009 | 2010 | 2011 | 2012 |
| 1 | 1.7 ± 0.6 (*58*) | 81.2 ± 2.8 (*52*) | 92.8 ± 1.1 (*54*) | 12.3 ± 3.2 (53) |
| 2 | 0.2 ± 0.2 (*48*) | 84.4 ± 1.9 (*48*) | 93.8 ± 1.0 (*47*) | 17.3 ± 3.3 (*45*) |
| 3 | 5.4 ± 1.8 (*26*) | 86.7 ± 3.0 (*30*) | 94.5 ± 1.3 (*29*) | 17.8 ± 4.8 (*24*) |
| 4 | 5.4 ± 1.6 (*26*) | 70.4 ± 2.9 (*25*) | 82.0 ± 2.4 (*25*) | 4.6 ± 1.3 (*24*) |
| 5 | 7.8 ± 1.3 (*72*) | 80.0 ± 2.5 (*70*) | 95.0 ± 1.0 (*68*) | 3.5 ± 0.6 (*68*) |
| 6 | 70.0 ± 4.4 (*29*) | 85.5 ± 3.0 (*29*) | 90.7 ± 2.0 (*29*) | 31.7 ± 4.7 (*29*) |
| 7 | 87.9 ± 2.5 (*63*) | 96.4 ± 1.0 (*64*) | 80.5 ± 3.0 (*64*) | 82.0 ± 3.2 (*64*) |
| 8 | 4.5 ± 1.6 (*22*) | 85.0 ± 3.2 (*22*) | 78.6 ± 2.6 (*22*) | 8.2 ± 1.7 (*22*) |
|  | mean ± SE (*N*) |  |  |  |
